# Viral Loads in Skin Samples of Patients with Monkeypox Virus Infection: A Systematic Review and Meta-Analysis

**DOI:** 10.3390/v15061386

**Published:** 2023-06-17

**Authors:** Isha Rani, Prakasini Satapathy, Anmol Goyal, Muhammad Aaqib Shamim, Amit Pal, Rosanna Squitti, Kalyan Goswami, Keerti Bhusan Pradhan, Sarvesh Rustagi, Alaa Hamza Hermis, Joshuan J. Barboza, Alfonso J. Rodriguez-Morales, Ranjit Sah, Bijaya K. Padhi

**Affiliations:** 1 Department of Biochemistry, Maharishi Markandeshwar College of Medical Sciences and Research (MMCMSR), Sadopur Ambala 134007, India; isha.rani@mmambala.org; 2 Global Center for Evidence Synthesis, Chandigarh 160036, India; contact@gces.network (P.S.); anmol.goyal@mmambala.org (A.G.);; 3 Department of Community Medicine, Maharishi Markandeshwar College of Medical Sciences and Research (MMCMSR), SadopurAmbala 134007, India; 4 Department of Pharmacology, All India Institute of Medical Sciences (AIIMS), Jodhpur 342001, India; 5 Department of Biochemistry, All India Institute of Medical Sciences (AIIMS), Kalyani 741250, India; amit.biochem@aiimskalyani.edu.in (A.P.); kalyan.biochem@aiimskalyani.edu.in (K.G.); 6 Department of Laboratory Science, Research and Development Division, Fatebenefratelli Isola Tiberina, Gemelli Isola, 00186 Rome, Italy; 7 Department of Healthcare Management, Chitkara School of Health Sciences, Chitkara University Punjab, Patiala 140401, India; keerti@chitkara.edu.in; 8 School of Applied and Life Sciences, Dehradun 180004, India; sarveshrustagi@uumail.in; 9 Nursing Department, Al-Mustaqbal University College, Hillah 51001, Iraq; alaa.hamza.hermis@uomus.edu.iq; 10 Escuela de Medicina, Universidad César Vallejo, Trujillo 13007, Peru; 11 Clinical Epidemiology and Biostatistics, School of Medicine, Universidad Científica del Sur, Lima 4861, Peru; alfonso.rodriguez@uam.edu.co; 12 Gilbert and Rose-Marie Chagoury School of Medicine, Lebanese American University, Beirut P.O. Box 36, Lebanon; 13 Tribhuvan University Teaching Hospital, Kathmandu 44600, Nepal; ranjitsah@iom.edu.np; 14 Department of Clinical Microbiology, Dr. D.Y. Patil Medical College, Hospital and Research Centre, Dr. D.Y. Patil Vidyapeeth, Pune 411000, India; 15 Department of Public Health Dentistry, Dr. D.Y. Patil Dental College and Hospital, Dr. D.Y. Patil Vidyapeeth, Pune 411018, India; 16 Department of Community Medicine and School of Public Health, Postgraduate Institute of Medical Education and Research, Chandigarh 160012, India

**Keywords:** monkeypox, skin swab, skin lesion, cutaneous, infection, meta-analysis

## Abstract

Despite monkeypox (mpox) being a public health emergency, there is limited knowledge about the risk of infectivity from skin viral loads during mpox infection. Thus, the aim of this study was to estimate cutaneous viral loads among mpox patients globally. Several databases, including Cochrane, EBSCOHost, EMBASE, ProQuest, PubMed, Scopus, and Web of Science, and preprint servers were searched concerning skin mpox viral loads in confirmed mpox subjects. In this systematic review and meta-analysis, a total of 331 articles were initially screened after the removal of duplicate entries. A total of nine articles were included in the systematic review and meta-analysis for the overall estimation of viral loads (Ct) using a random-effect model. The pooled cutaneous mpox viral load (lower Ct) was 21.71 (95% CI: 20.68–22.75) with a majority of positivity rates being 100%, highlighting a higher infectivity risk from skin lesions. The current results strongly support that skin mpox viral loads may be a dominant source of rapid transmission during current multi-national outbreaks. This important finding can help in constructing useful measures in relevant health policy.

## 1. Introduction

Human monkeypox (mpox) is a viral zoonotic disease caused by the mpox virus and is closely related to the variola virus, which causes smallpox [1]. It was first identified in humans in 1970 in the Democratic Republic of Congo (DRC) and is currently found primarily in Central and West Africa [2]. The clinical presentation of mpox is similar to that of smallpox, but it is generally less severe [3]. Despite being rare, it has a high mortality rate, and there is no adequate test, specific treatment, or vaccine for it [4]. There are 87,078 total confirmed cases and 130 total deaths of mpox reported worldwide as of 26 April 2023, according to Centers for Disease Control and Prevention (CDC) data [5]. Till now, there have been confirmed cases in more than 100 member states across all six World Health Organization (WHO) regions. The WHO declared mpox outbreaks a potential “Public Health Emergency of International Concern (PHEIC)” on 23 June 2022 [6].

**Figure 1:**
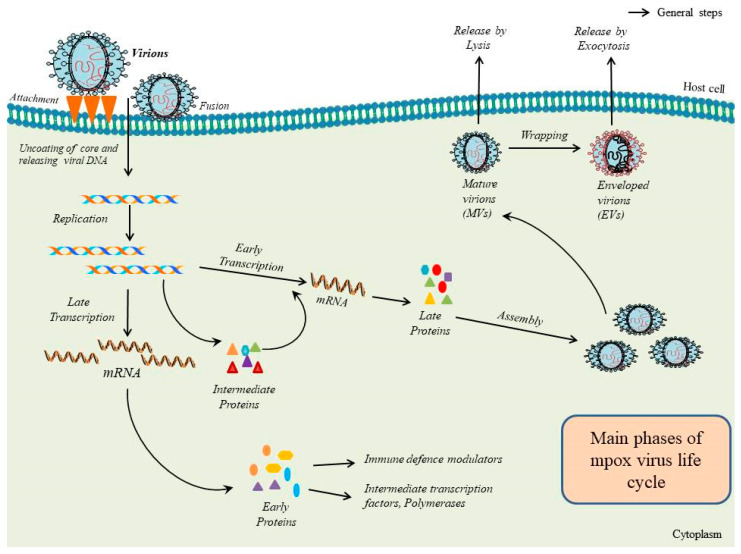
A detailed representative image showing the main phases of the life cycle of monkeypox (mpox) virus inside a human cell.

Mpox is a large oval-shaped virus with an average size ranging from 200 to 250 nm and contains a core area with lateral bodies, double-stranded deoxyribonucleic acid (dsDNA) and a lipoprotein envelope. The nucleocapsid core of the mpox virus is biconcave with a lateral body on both sides, as visualized using electron microscopy. The outer core of the mpox virus includes the epitopes of antigens that permit the virus’s entry into the host cell. Mpox contains linear-shaped dsDNA (197 kb; 197,205 bp) with covalently closed hairpin ends (no free 3′ and 5′ ends) and contains 190 non-overlapping open reading frames (ORFs). The central coding region sequence (CRS) is highly conserved and flanked by variable inverted terminal repeats (ITR; 10 kb) located at both ends of the genome. The “housekeeping” genes responsible for the main phases of the life cycle, such as transcription, replication and virion assembly, are present in the conserved central area. However, the genes in the terminal domains may be altered according to diverse poxviruses and create proteins concerned in ailments and the host range [7,8]. Mpox carries out the main phases of its life cycle in an infected cell’s cytoplasm. During viral attachment, a virion enters within the host cells by binding and fusing with the host cell membrane. The viral core is released into the cytoplasm, and viral particles are assembled into intracellular mature virions (MVs). These intracellular MVs reside in the cytoplasm and are released during cell lysis. However, MVs can attain an additional envelope by wrapping and then release as extracellular enveloped virions (EVs) via exocytosis (Figure 1) [8]. Mpox symptoms are generally characterized by fever, flu-like signs, skin lesions/rash and lymphadenopathy. However, mpox might even be asymptomatic or have atypical clinical presentations [9,10,11,12,13,14]. Most of the patients suffer from a moderate form of the mpox illness and do not require hospitalization or antiviral therapy. However, serious mpox complications comprise pneumonitis, secondary bacterial infections, keratitis, encephalitis, myocarditis, epiglottitis and soft-tissue infections such as cellulitis and abscesses [9,15,16]. Importantly, mpox virus transmission occurs directly or indirectly via large respiratory droplets, close or direct contact with skin lesions, bodily fluids and, probably, contaminated fomites [11].

Viral load is an important indicator of the severity of infection, and it has been shown that higher viral loads are associated with more severe disease. Numerous studies have investigated viral burden in the skin samples of patients with mpox [11,17,18] and indicated that viral loads are higher in patients with severe disease and in children than in adults [9,19]. Based on this evidence, it is expected that skin samples may represent a great reservoir of mpox virus. Consistently, a growing body of evidence suggests that viral loads in skin samples can be used as a biomarker for the severity of mpox infection. The determination of viral loads in skin samples could be predictive of the severity and prognosis of the infection. Establishing whether the viral load is predictive of the disease severity might benefit treatment development, the setting up of strategies or even put into place actions for disease control.

Importantly, skin samples can be used as an easily accessible source of the virus and are less invasive than other samples such as blood or cerebrospinal fluid. Additionally, skin samples can be collected at different stages of the infection, allowing the determination of changes in viral load over time. Moreover, factors that influence the viral load, including age, sex, and the overall health conditions of the patients, can be investigated, in addition to the determination of the potential impact of differences in regions or populations. The high number of patients populating modern hospitals and organizational and bureaucratic problems often make it relatively difficult for scholars to recruit suitable numbers of individuals for enrollment in clinical studies. In fact, it is usual to see published studies on widespread clinical conditions based on relatively small and frequently heterogeneous samples. This has a negative impact on the power and reliability of the results. Meta-analysis, a statistical technique that combines the results of different studies addressing a specific topic, can mitigate these issues and define whether variations in effect among studies are due to different analytical approaches; differences in the samples, including sex, age, ethnicity; or random statistical fluctuation. The estimated ‘true effect size’ is the outcome of this type of analysis and increases the power of the conclusions. In this paper, we applied a systematic review and a meta-analysis to major studies published till 17 January 2023 on viral loads in the skin samples of patients with mpox virus infection. The outcome of this study can provide valuable insights into the disease and improve the advancements of effective therapeutic actions that can be put in place to contain the spread of the disease. This new knowledge may be beneficial to curtail the spread of this endemic infection.

## 2. Methods

### 2.1. Search Strategy and Selection Criteria

The search strategy of this systematic review and meta-analysis was designed according to PECOS criteria (refer to Supplementary Files, Annexure S3) and was based on the following: “what is the mpox viral load in skin specimens among patients infected with mpox?”. The participants confirmed with mpox virus infection by real-time polymerase chain reaction (PCR) were included in this study. The confirmed cases were selected regardless of age and gender for inclusion criteria. On the other hand, suspected or probable subjects with mpox infection were excluded.

The protocol concerning this systematic review and meta-analysis was registered on PROSPERO (CRD42023392505). The present study followed the Meta-Analysis of Observational Studies in Epidemiology (MOOSE) reporting guidelines [20] and the 2020 Preferred Reporting Items for Systematic Reviews and Meta-Analysis (PRISMA) guidelines [21]. A systematic search was conducted on Cochrane Library, EBSCOHOST, EMBASE, ProQuest, PubMed/MEDLINE, Scopus and Web of Science from the inception of each database till 17 January 2023 according to PECOS criteria (see Supplementary Files, Annexure S3 for search strategy and Annexure S2 for PECOS criteria). Medical subject headings (MeSH) and truncated terms with an asterisk were also used to identify potentially eligible articles. Preprint servers such as bioRxiv and medRxiv were also screened. In addition to this, we manually explored the references of relevant articles as well as Google Scholar and Google for additional studies missed out during the electronic search. This systematic review ensured a broad scope, including subsequent observational studies such as cross-sectional, cohort and case series studies. Additionally, studies evaluating the mpox viral loads in skin samples of confirmed monkeypox participants regardless of age and gender were included. It is important to mention that relevant communications, reports and editorials that presented some data from case series studies with greater than ten cases were also included. However, studies without direct relevance to the present objective as well as those that were only abstracts, qualitative, policy, case reports, reviews and opinion reports were excluded. The 629 articles resulting from the electronic search were further exported and saved to the data management software Mendeley desktop V1.19.5 software to manage citations, remove duplicate entries and randomized controlled trials (RCTs), and synchronize the review process.

### 2.2. Inclusion and Exclusion Criteria

The inclusion criteria were based on mpox-confirmed patients regardless of country, age, race and gender. All articles published till 17 January 2023 were considered for this study (Supplementary Files, Annexure S2). The exclusion criteria were considered on the basis of suspected or probable cases of mpox. Any unpublished data, abstracts, preceding papers, case reports and articles without available full texts were excluded.

### 2.3. Data Extraction and Management 

Two independent authors (IR and AG) went through the title and abstracts of the obtained studies by applying the eligibility criteria and selected 17 articles for full-text screening. If there was any conflict or discrepancy for the inclusion of a study for full-text assessment, the co-authors communicated among themselves to maintain harmony and decided unanimously on the admissibility. A third author (MAS) decided the unsolved ambiguities. Data extraction was performed by two independent authors (IR and AG). In the case of disparity at any stage, the authors discussed among themselves to decide inclusion. The third author (MAS) decided the unsolved ambiguities. Further, data were extracted in a Microsoft Excel spreadsheet for further analysis. It is worth mentioning that in studies in which the mean value was not available, the data were reconstructed from box plots with WebPlotDigitizer tool (Version 4.6) [22] and then calculated using other protocols [23,24]. The approximate mean and standard deviation was observed using median and interquartile range (IQR) after testing for skewness [25].

The subsequent information assembled from each of the final eligible articles is explained in the following manner: the author’s name; the year of publication; the study design; the number of mpox participants from whom skin samples were taken; the prevalence of mpox DNA in skin samples; age; gender; the regions where the study was conducted.

**Figure 2:**
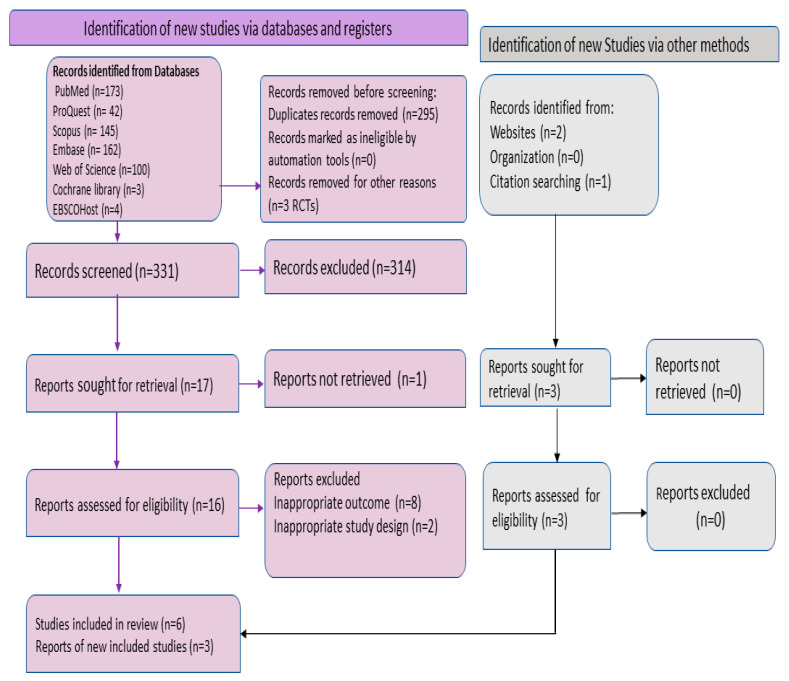
PRISMA (Preferred Reporting Items for Systematic Reviews and Meta-Analysis) flowchart summarizing the literature search and giving reasons for exclusion of studies.

The whole procedure of the literature search, screening, data extraction, systematic review and meta-analysis was described using the Preferred Reporting Standard of Systematic Reviews and Meta-Analysis (PRISMA-2020) flowchart and checklist to ensure scientific accuracy (Figure 2; Supplementary Files, Annexure S1 for PRISMA checklist). 

### 2.4. Quality Assessment

Two independent authors (IR and AG) evaluated the risk of bias in the included studies using the quality assessment tools recommended by National Institute of Health (NIH) [26]. The case series, cross-sectional and cohort studies were assessed using NIH quality assessment tool. Any differences between the authors related to the risk of bias in any of the studies were solved by conversation. The third author (MAS) decided the unsolved ambiguities. The rating and overall score for each study are given in the Supplementary Files, Annexure S4. 

### 2.5. Statistical Analysis

According to all included studies, the mpox viral loads in skin samples are represented as ‘Ct’ (mean ± SD). Here, Ct is defined as the PCR cycle threshold, which is necessary to multiply the viral genetic material to a detectable limit and has been used as a representative for viral loads and infectivity. We extracted data on the Ct value of skin samples positive for mpox. Then, we pooled this data from all the studies to arrive at the average Ct value of mpox-positive skin samples. We employed an inverse variance method with random study effects. We visualized the results in the form of a forest plot. We assessed heterogeneity using Cochran’s Q, tau-squared and prediction interval. Maximal likelihood estimator computed the confidence interval for tau-squared [27]. Prediction interval is a more practical estimator of heterogeneity in that it gives a range of values into which the result of a similar original study in the future is expected to lie [28]. Heterogeneity and influence of individual studies was also assessed by clustering, Baujat plot and influence plots. We explored potential sources of heterogeneity of categorical variables using subgroup analyses and quantitative variables using meta-regression. Bubble plots were used to visualize the results of meta-regression. We conducted sensitivity analyses by omitting potential outliers and overly influential studies and analyzed each study individually. For assessment of publication bias and small-study effects, we used a funnel plot. We carried out influence analysis by assessing the effect of leaving out studies one-by-one on the overall effect size. All statistical analyses were conducted using meta, metafor and dmetar package in R programming language (v4.2.2) [29]. *p* < 0.05 (two-sided) was considered statistically significant.

## 3. Results

### 3.1. Selection Criteria and Baseline Characteristics of Included Studies

The systematic search generated 629 articles, among which 295 duplicates and 3 RCTs were detected. The title/abstract screening of the 331 articles was performed, and 314 articles were observed to be ineligible and eliminated because they did not meet the inclusion criteria of this study. Full-text screening was performed on 16 articles, and the full text of 1 article was not retrieved after considering eligibility among them [30]. After full-text screening, 10 articles that did not satisfy the eligibility criteria were excluded. One of the reasons was the failure of the average Ct values to meet the inclusion criteria not mentioned in these studies [11,31,32,33,34]. Moreover, three other articles were found to be eligible because of relevant citations of included studies and websites. A total of nine studies that were included in the meta-analysis concerned overall pooled mpox viral loads (Ct) in skin specimens (Table 1).

The PRISMA flowchart illustrates the selection process of the articles (Figure 2). The quality assessment of the included studies is depicted in the supplementary data (Annexures S4a,b). All nine studies were found to be of good quality. The inclusion of the studies was conducted till 17 January 2023. The baseline characteristics of nine studies included in the systematic review and meta-analysis, including one case series [17], one cross-sectional study [35], six prospective observational studies [18,36,37,38,39,40] and one retrospective cohort study [41], are explained in Table 1. All studies were conducted in countries where mpox is not endemic, such as Spain (4/9, 44.44%), France (3/9, 33.33%), Italy (1/9, 11.11%) and Canada (1/9, 11.11%). Most of the cases were in adults older than 18 years. The sample size of the included studies ranged from 10 [39] to as high as 258 [38]. Furthermore, four out of nine studies reported a travel history in mpox participants [18,38,39,40], in values ranging from 14.3% to 80%. In all the included studies, mpox was confirmed by diagnostic testing such as real-time PCR for mpox DNA. Additionally, most of the cases were found in males and mostly consisted of MSM (men who have sex with men). In the prospective observational study with the largest sample size by Mailhe M et al., 99.2% (256 out of 258) of mpox-confirmed cases were in MSM [38]. The frequent systemic symptoms or complaints presented by the majority of mpox patients included rash, fever, headaches, myalgia, fatigue and lymphadenopathy, while others included tonsillitis, proctitis, pharyngitis, odynophagia, epiglottitis and asthenia. The highest mpox viral loads (lower mean value Ct; PCR cycle thresholds) in skin samples were observed in the studies conducted in Northern France (Ct: 19.5 ± 4.7; mpox DNA positivity rate: 100%) [35] and Paris, France (Ct: 20 ± 2; mpox DNA positivity rate: 88%) [17], while the lowest viral loads (higher Ct mean value) were identified in other regions of France (Ct: 23.4 ± 3.7; mpox DNA positivity rate: 100%) [38] and Ontario, Canada (Ct: 23.1 ± 6.5; mpox DNA positivity rate: 43.6%) [41].

**Table 1:** Baseline characteristics of included studies that reported mpox viral DNA (based on Ct values) in skin samples of patients with mpox (N = 9 studies).

Authors (YOP)	Study Design	Ct Mean (±SD)	Number of Mpox-Confirmed Cases from Whom Skin Samples Were Taken	Prevalence of Mpox Viral DNA in Skin Samples (%)	Age (Years) Median	Gender	Region
Hasso M et al., (2022) [41]	RC	23.1 (6.5)	78	43.60%	38 *	All males	Ontario, Canada
Loconsole D et al., (2022) [39]	PO	21.2 (5.4)	10	100%	36.7	8 males (6 MSM) and 2 females	Southern Italy
Mailhe M et al., (2022) [38] ^#^	PO	23.4 (3.7)	258	98%	35	Majority of males, including MSM, except 1 female and 1 transgender female	France
Ouafi M et al., (2022) [35] ^#^	COS	19.5 (4.7)	116	100%	37	All males (including mostly MSM), except 1 female	Northern France
Palich R et al., (2023) [17] ^#^	CS	20 (2)	50	88%	34	All males (49 MSM and 1 MSW)	Paris, France
Peiró-Mestres A et al., (2022) [18]	PO	20.5 (3.1)	12	100%	38.5	All males (MSM)	Barcelona, Spain
Tarín-Vicente EJ et al., (2022) [40]	PO	23 (4)	180	99%	37	Majority males, including gay, bisexual, and MSM, except for a few heterosexual males or females	Madrid and Barcelona, Spain
Ubals M et al., (2022) [36] ^#^	PO	22.5 (2.9)	49	100%	33.5	All males	Spain
Veintimilla C et al., (2022) [37]	PO	21.8 (4.6)	37	97%	31	All males (MSM)	Madrid, Spain

YOP: year of publication; Ct: polymerase chain reaction (PCR) cycle threshold; SD: standard deviation; DNA: deoxyribonucleic acid; MSM: men who have sex with men; MSW: men who have sex with women; %: percentage; RC: retrospective cohort; PO: prospective observational; COS: cross-sectional; CS: case series. Here, Ct values are represented as Mean ± SD; ^#^ denotes studies in which mean and SD of Ct were not available, and mean values of Ct were calculated from box plots using WebPlotDigitizer tool (Version 4.6) [22] and then calculated using other protocols [23,24]. The approximate mean and SD were estimated using median and interquartile range (IQR) after testing for skewness [25]. Age (years) is represented in median; * represents mean value.

### 3.2. Pooled Prevalence

**Figure 3:**
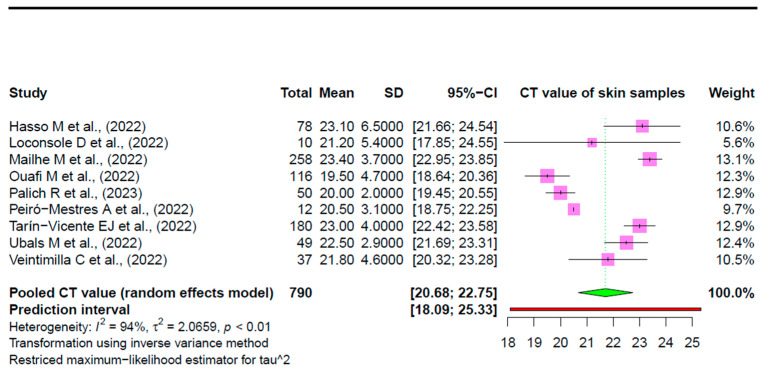
Forest plot summarizing the individual studies and pooled estimate of viral loads in skin samples of patients with mpox based on Ct values. The green diamond represents the pooled estimate according to the random effects model. The pink boxes represent the estimates from the individual studies. The red line represents the prediction interval [17,18,35,36,37,38,39,40,41].

All nine studies were included in the meta-analysis to estimate the pooled viral loads (Ct) from cutaneous samples taken from 790 out of 823 mpox-confirmed individuals. The pooled mean Ct value of skin samples positive for mpox is 21.71 (95% CI: 20.68–22.75) (Figure 3). The estimates from the nine individual studies ranged from 19.50 [35] to 23.40 [38].

### 3.3. Heterogeneity Estimation and Exploration

Individual studies showed heterogeneity with an *I*^2^ of 94% and a tau-squared of 2.07. The prediction interval ranged from 18.09 to 25.33 (Figure 3). To reduce this heterogeneity, we performed subgroup analysis and meta-regression. 

Subgrouping the studies based on country significantly reduced heterogeneity (*p* = 0.004) (Table 2).

**Figure 4:**
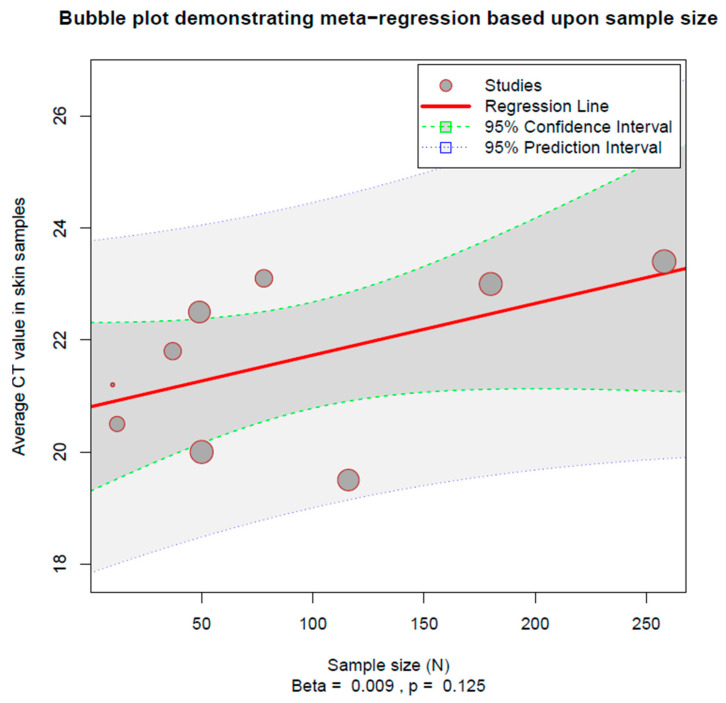
Bubble plot demonstrating meta-regression based upon the size of the study.

We conducted meta-regression based upon the sample size of individual studies and the average age of participants. Neither was significant (sample size: beta = 0.009, *p* = 0.125, Figure 4; average age: beta = −0.038, *p* = 0.876, Supplementary Files, Annexure S5; sex distribution: beta = −0.505, *p* = 0.966, Supplementary Files, Annexure S6).

### 3.4. Influence Assessment

**Figure 5:**
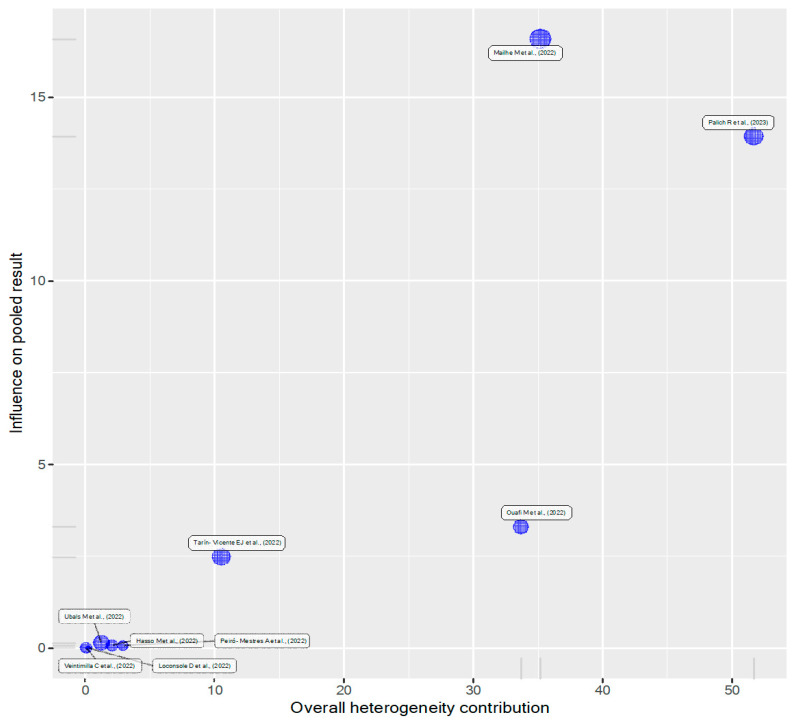
Baujat plot to identify studies contributing overly to influence and heterogeneity [17,18,35,36,37,38,39,40,41].

We used a Baujat plot, influence plots and clustering to identify studies with a high influence. The Baujat plot (Figure 5) and clustering (refer to Supplementary Files, Annexure S7a–d) show three studies [17,35,38] to exert a high influence on the overall result and contribute considerably to the overall heterogeneity. The influence plots did not flag any study (Annexure S7e). The overall heterogeneity can be assessed visually here (Annexure S7f).

### 3.5. Sensitivity Analysis

**Figure 6:**
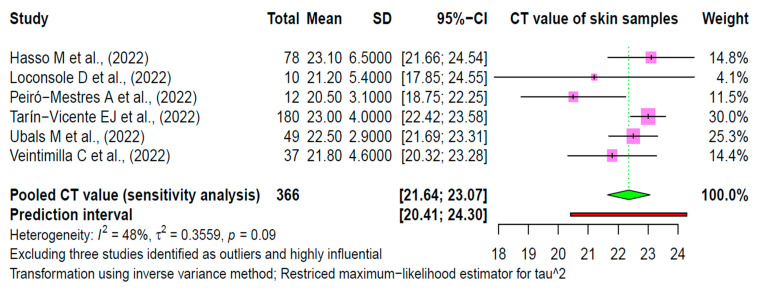
Sensitivity analysis after omitting overly influential studies. The green diamond represents the pooled estimate according to the random effects model. The pink boxes represent the estimates from the individual studies. The red line represents the prediction interval [17,18,35,36,37,38,39,40,41].

We performed a sensitivity analysis omitting three highly influential studies contributing significantly to heterogeneity. The pooled estimate was increased from 21.71 (95% CI: 20.68–22.75) to 22.36 (95% CI: 21.64–23.07) with an *I*^2^ of 48% (earlier 94%) (Figure 6). None of the studies were of low quality, and, hence, no sensitivity analysis was conducted for the quality of studies. We also performed a leave-one-out meta-analysis, omitting each individual study one-by-one. *I*^2^ did not vary much, ranging from 90% to 95% (Supplementary Files, Annexure S8).

### 3.6. Publication Bias and Small-Study Effects

We constructed a funnel plot (Supplementary Files, Annexure S9). Upon visual inspection, there might seem some evidence of publication bias. We could not quantify it since Egger’s regression cannot be used when there are less than ten studies. However, we should be careful as funnel plots are suited for two-group or comparative meta-analysis.

## 4. Discussion

To the best of our knowledge, the current systematic review and meta-analysis (SRMA) is the first study to estimate overall viral burden in skin samples of mpox-infected cases. This SRMA included 823 total confirmed patients with mpox infection from nine studies published globally till 17 January 2023. The cutaneous samples were taken for viral load analysis from 790 cases out of 823 total confirmed patients. 

The pooled skin viral load among patients with mpox infection was observed to be 21.71 (95% CI: 20.68–22.75) from 731 out of 790 confirmed mpox individuals from nine studies.

This SRMA revealed a high mpox positivity rate and lower Ct values in the skin specimens of mpox-confirmed cases. There is an inverse relationship between Ct values and viral loads. Hence, lower Ct values represent high viral loads and infectivity [42,43,44]. In this study, the high prevalence of mpox viral loads (lower Ct) and mpox’s positivity rate in cutaneous specimens is suggestive of increased infectivity from direct contact with skin lesions.

In corroboration with this, a significant correlation was observed between viral DNA levels in skin specimens and infectivity in BSC-1 cell lines (exhibiting an epithelial morphology), which is validated by the higher viral DNA content (lower Ct values) in skin lesions of most of the patients evaluated. Notably, this strongly predicts high infectivity in cell lines (BSC-1) and indicates a higher risk of infectivity from dermal lesions. Moreover, an mpox-infected patient with lesions is regarded as infectious till the crust from crusty lesions falls off [35]. The samples with a Ct value ≥ 35 are considered poor or non-infectious samples [42]. Similarly, Lapa and colleagues reported that patients with a Ct value of 29 in semen samples successfully recovered from mpox infection [45]. Similarly, Hernaez et al. observed that the increase in infectious mpox virus was most likely higher in saliva samples with Ct values < 26 [44].

The present results confirm the previous findings from a meta-analysis showing a high mpox DNA positivity rate in skin samples among other different clinical samples [46]. The current data extends the previous findings collected from a small subset of studies published till August 2022. By analyzing quantitative results on a large subset of studies available worldwide till 17 January 2023, we confirmed the presence of high mpox viral loads in skin specimens more effectively. Furthermore, the current investigation significantly validated higher mpox viral loads (lower Ct) in dermal specimens on the basis of recent multiple studies available worldwide. Notably, Noe and colleagues have observed the highest viral load in the skin swabs of the first two mpox patients in Germany [47].

In support of our data, a longitudinal study of viral DNA load kinetics revealed the reliability of a higher mpox viral load in skin lesion swabs, which was observed at the late stages of the disease. This study also reported consistently high viral DNA loads at or above 10^6^ copies/mL in swabs of skin lesions during the initial and serial testing. Despite this, all skin lesion swabs were positive for mpox viral DNA during the entire time course as compared to oropharyngeal samples [34], suggesting that contact transmission via mpox skin lesions may be a dominant route of mpox infection. 

These findings highlight that skin lesion swabs are the best type of specimens for the diagnosis of mpox infection: they can be easily assessed by using real-time PCR and yield a high viral burden and positivity rate. In line with these results, the WHO has defined skin lesions as the preferred diagnostic specimens for laboratory mpox confirmation [48]. This is because antigen or antibody detection methods can not differentiate between orthopoxviruses; hence, real-time-PCR-based diagnostics can be considered to be more specific [49]. Above all, samples are easy to preserve and collect from the roof or fluid of the vesicles, pustules and dry crusts of the skin lesions. Consistently, a recent study compared self- and physician-collected samples and observed no significant differences in viral loads (Ct) by both procedures in skin swabs as compared to rectal swabs [36]. This suggests that skin samples are the most feasible and accurate source for self-sampling and undoubtedly offer potential benefits for patients and disease control. Furthermore, our study suggests that the main route for virus transmission is direct cutaneous contact. This implies that prevention and containment measures can be put in place to contain the spread of the infection in multinational outbreaks.

Importantly, this study has some limitations. First, in this current study, the pooling of skin viral loads is based on a limited sample size and relevant studies. Ideally, a larger sample size would grant good precision in data estimation. However, the current outcomes are based on many studies, and, thus, the current study seems reliable. Further, the correlation of skin viral loads with a high risk of infectivity is based upon the specific characteristics of mpox infections. For example, most of the included studies had male mpox cases of all ages from mainly non-endemic regions. It would be a better approach to underscore the role of cutaneous viral loads in the severity and infectivity of disease according to age, gender and endemic and non-endemic regions. Another limitation consists of the lack of information about mucocutaneous samples or body fluids as potential routes of direct contact transmission. In the current study, the pooled mean value of Ct for mpox viral DNA in mpox positive samples is 21.71 (95% CI: 20.68–22.75), and the values are expected to lie within a prediction interval of 18.09 to 25.33. The studies were highly heterogeneous with an *I*^2^ of 94%. This value does not vary with age, sex distribution or sample size. Eliminating three highly influential studies contributing to heterogeneity reduced the heterogeneity from *I*^2^ = 94% to *I*^2^ = 48%, and the average Ct value increases slightly from 21.71 (95% CI: 20.68–22.75) to 22.36 (95% CI: 21.64–23.07).

## 5. Conclusions

Altogether, the present study provides information regarding the role of direct skin-to-skin contact in mpox transmission, revealing its high risk of infectivity. Based on these results, it would be beneficial to put in place awareness measures for the prevention or containment of mpox spread. The public should be aware of frequent transmission via skin contact or physical promiscuity during social events.

## Figures and Tables

**Table 2:** Subgroup analyses based on countries.

Result for Subgroup Differences (Common Effect Model)						
Country	k	Mean	95% CI		Q	*I^2^*
Canada [41]	1	23.1000	[21.6575; 24.5425]		0.00	--
Italy [39]	1	21.2000	[17.8531; 24.5469]		0.00	--
France [17,35,38]	3	21.6791	[21.3551; 22.0031]		115.99	98.3%
Spain [18,36,37,40]	4	22.5948	[22.1574; 23.0323]		8.48	64.6%
**Test for Subgroup Differences (Common Effect Model)**						
**Between groups**		**Q.d.f**		***p* value**		
13.26		3		0.0041		

## Supplementary Materials

The following supporting information can be downloaded at: https://www.mdpi.com/article/10.3390/v15061386/s1, Annexure S1: PRISMA checklist; Annexure S2: Inclusion and exclusion criteria (according to PECOS); Annexure S3: The adjusted search terms as per the PECOS framework (Viral loads in skin samples of patients with monkeypox virus infection): searched electronic databases [as of 17.01.2023]; Annexure S4: Quality assessment of included cross-sectional studies and cohort studies with the use of NIH tools: (a) for Case series; (b) for Cross-sectional studies; Annexure S5: Bubble plot demonstrating meta-regression based upon the average age of the participants; Annexure S6: Bubble plot demonstrating meta-regression based upon the sex distribution of the participants; Annexure S7 (a–d): Clustering to identify influence and heterogeneity contributed by studies; (e) Influence plot to visualise influence; (f) Visualisation of heterogeneity contributed by the studies plotted against pooled estimate; Annexure S8: Leave-one-out meta-analysis for each study showing the effect on the pooled estimate; Annexure S9: Funnel plot to demonstrate publication bias and small-study effects.
