# Supplementary material for: Viral Loads in Skin Samples of Patients with Monkeypox Virus Infection: A Systematic Review and Meta-Analysis
Source: Viruses. 2023 Jun 17;15(6):1386. doi: 10.3390/v15061386 (PMC10301466; doi:10.3390/v15061386)

List of Supplementary files Captions

Annexure S1: PRISMA checklist

Annexure S2: Inclusion and exclusion criteria (according to PECOS)

Annexure S3: The adjusted search terms as per the PECOS framework (Viral loads in skin samples of patients with monkeypox virus infection): searched electronic databases [as of 17.01.2023]

Annexure S4: Quality assessment of included cross-sectional studies and cohort studies with the use of NIH tools: *a) for Case series; b) for Cross-sectional studies*

Annexure S5: Bubble plot demonstrating meta-regression based upon the average age of the participants

Annexure S6: Bubble plot demonstrating meta-regression based upon the sex distribution of the participants

Annexure S7 (a-d): Clustering to identify influence and heterogeneity contributed by studies; **e)** Influence plot to visualise influence; **f)** Visualisation of heterogeneity contributed by the studies plotted against pooled estimate

Annexure S8: Leave-one-out meta-analysis for each study showing the effect on the pooled estimate

Annexure S9: Funnel plot to demonstrate publication bias and small-study effects

Annexure S1: PRISMA checklist

Section and Topic	Item #	Checklist item	Location where item is reported
**TITLE**			
Title	1	Identify the report as a systematic review.	1
**ABSTRACT**			
Abstract	2	See the PRISMA 2020 for Abstracts checklist.	3
**INTRODUCTION**			
Rationale	3	Describe the rationale for the review in the context of existing knowledge.	6
Objectives	4	Provide an explicit statement of the objective(s) or question(s) the review addresses.	6
**METHODS**			
Eligibility criteria	5	Specify the inclusion and exclusion criteria for the review and how studies were grouped for the syntheses.	6
Information sources	6	Specify all databases, registers, websites, organisations, reference lists and other sources searched or consulted to identify studies. Specify the date when each source was last searched or consulted.	7
Search strategy	7	Present the full search strategies for all databases, registers and websites, including any filters and limits used.	6,7
Selection process	8	Specify the methods used to decide whether a study met the inclusion criteria of the review, including how many reviewers screened each record and each report retrieved, whether they worked independently, and if applicable, details of automation tools used in the process.	6-8
Data collection process	9	Specify the methods used to collect data from reports, including how many reviewers collected data from each report, whether they worked independently, any processes for obtaining or confirming data from study investigators, and if applicable, details of automation tools used in theprocess.	8
Data items	10a	List and define all outcomes for which data were sought. Specify whether all results that were compatible with each outcome domain in each study were sought (e.g. for all measures, time points, analyses), and if not, the methods used to decide which results to collect.	8
	10b	List and define all other variables for which data were sought (e.g. participant and intervention characteristics, funding sources). Describe any assumptions made about any missing or unclear information.	8
Study risk of bias assessment	11	Specify the methods used to assess risk of bias in the included studies, including details of the tool(s) used, how many reviewers assessed each study and whether they worked independently, and if applicable, details of automation tools used in the process.	9
Effect measures	12	Specify for each outcome the effect measure(s) (e.g. risk ratio, mean difference) used in the synthesis or presentation of results.	9

Synthesis methods	13a	Describe the processes used to decide which studies were eligible for each synthesis (e.g. tabulating the study intervention characteristics and comparing against the planned groups for each synthesis (item #5)).	Annexure 2
	13b	Describe any methods required to prepare the data for presentation or synthesis, such as handling of missing summary statistics, or data conversions.	8
	13c	Describe any methods used to tabulate or visually display results of individual studies and syntheses.	8-9
	13d	Describe any methods used to synthesize results and provide a rationale for the choice(s). If meta-analysis was performed, describe the model(s), method(s) to identify the presence and extent of statistical heterogeneity, and software package(s) used.	8-9
	13e	Describe any methods used to explore possible causes of heterogeneity among study results (e.g. subgroup analysis, meta-regression).	9
	13f	Describe any sensitivity analyses conducted to assess robustness of the synthesized results.	N/A
Reporting bias assessment	14	Describe any methods used to assess risk of bias due to missing results in a synthesis (arising from reporting biases).	9
Certainty assessment	15	Describe any methods used to assess certainty (or confidence) in the body of evidence for an outcome.	9

Section and Topic	Item #	Checklist item	Location where item is reported
**RESULTS**			
Study selection	16a	Describe the results of the search and selection process, from the number of records identified in the search to the number of studies included in the review, ideally using a flow diagram.	Fig 1
	16b	Cite studies that might appear to meet the inclusion criteria, but which were excluded, and explain why they were excluded.	N/A
Study characteristics	17	Cite each included study and present its characteristics.	Table 1
Risk of bias in studies	18	Present assessments of risk of bias for each included study.	Annexure 4
Results of individual studies	19	For all outcomes, present, for each study: (a) summary statistics for each group (where appropriate) and (b) an effect estimate and its precision (e.g. confidence/credible interval), ideally using structured tables or plots.	Fig 2
Results of syntheses	20a	For each synthesis, briefly summarise the characteristics and risk of bias among contributing studies.	Annexure 4
	20b	Present results of all statistical syntheses conducted. If meta-analysis was done, present for each the summary estimate and its precision (e.g. confidence/credible interval) and measures of statistical heterogeneity. If comparing groups, describe the direction of the effect.	Fig 2
	20c	Present results of all investigations of possible causes of heterogeneity among study results.	Fig 4
	20d	Present results of all sensitivity analyses conducted to assess the robustness of the synthesized results.	N/A
Reporting biases	21	Present assessments of risk of bias due to missing results (arising from reporting biases) for each synthesis assessed.	N/A
Certainty of evidence	22	Present assessments of certainty (or confidence) in the body of evidence for each outcome assessed.	11-12
**DISCUSSION**			
Discussion	23a	Provide a general interpretation of the results in the context of other evidence.	13

	23b	Discuss any limitations of the evidence included in the review.	15
	23c	Discuss any limitations of the review processes used.	N/A
	23d	Discuss implications of the results for practice, policy, and future research.	16
**OTHER INFORMATION**			
Registration and protocol	24a	Provide registration information for the review, including register name and registration number, or state that the review was not registered.	6
	24b	Indicate where the review protocol can be accessed, or state that a protocol was not prepared.	6
	24c	Describe and explain any amendments to information provided at registration or in the protocol.	N/A
Support	25	Describe sources of financial or non-financial support for the review, and the role of the funders or sponsors in the review.	1
Competing interests	26	Declare any competing interests of review authors.	1
Availability of data, code and other materials	27	Report which of the following are publicly available and where they can be found: template data collection forms; data extracted from included studies; data used for all analyses; analytic code; any other materials used in the review.	2

Annexure S2: Inclusion and exclusion criteria according to PECOS

Participants	Exposure	Comparator	Outcome	Study design
**Inclusion criteria:**<br>All confirmed mpox patients<br>• All ages<br>• All gender<br><br>**Exclusion criteria:**<br>Suspected or probable mpox patients	Not applicable	Not applicable	**Inclusion criteria:**<br>mpox viral loads (Ct) in skin samples	**Inclusion criteria:**<br>Observational studies, Prevalence studies, case series, cross-sectional studies, cohort studies, case control studies, surveys, preprints, Editorial with case series data, Rapid or Short communication with case series data, brief reports with case series data.<br>Geography-Global level.<br>Date of Search- Published till 17th January 2022. English Language. Human studies.<br>**Exclusion criteria:**<br>Qualitative, Policy, case reports, Opinion reports, abstracts

Annexure S3: The adjusted search terms as per the PECOS framework [Viral loads in skin samples of patients with monkeypox virus infection]: searched electronic databases [as of 17.01.2023]

Database	No	Search Query	Results
**Cochrane**			
	#1	(mpox:ti,ab) OR (monkeypox:ti,ab) OR (mpxv:ti,ab)	14
	#2	(skin:ti,ab) OR (cutaneous*:ti,ab)	61,030
	#3	(lesion*:ti,ab) OR (swab:ti,ab) OR (sample:ti,ab)	145,138
	#4	#1 AND #2 AND #3	3
**EBSCOHost-Academic Search Complete**			
	#1	((TI mpox OR AB mpox)) OR ((TI monkeypox OR AB monkeypox)) OR ((TI mpxv OR AB mpxv))	104
	#2	((TI skin OR AB skin)) OR ((TI cutaneous* OR AB cutaneous*))	19,016
	#3	((TI lesion* OR AB lesion*)) OR ((TI swab OR AB swab)) OR ((TI sample OR AB sample))	134,813
	#4	S1 AND S2 AND S3	4
**EMBASE**			
	#1	(mpox:ti,ab) OR (monkeypox:ti,ab) OR (mpxv:ti,ab)	2,577
	#2	(skin:ti,ab) OR (cutaneous*:ti,ab)	985,816
	#3	(lesion*:ti,ab) OR (swab:ti,ab) OR (sample:ti,ab)	2,660,413
	#4	#1 AND #2 AND #3	162
**ProQuest**			
	#1	(TI,AB(mpox)) OR (TI,AB(monkeypox)) OR (TI,AB(mpxv))	564
	#2	(TI,AB(skin)) OR (TI,AB(cutaneous*))	109,047
	#3	(TI,AB(lesion*)) OR (TI,AB(swab)) OR (TI,AB(sample))	1,135,291
	#4	1 AND 2 AND 3	42
**PubMed**			
	#1	(mpox[Title/Abstract]) OR (monkeypox[Title/Abstract]) OR (mpxv[Title/Abstract]) OR ("monkeypox"[MeSH])	2,457
	#2	("skin"[MeSH Terms]) OR (skin[Title/Abstract]) OR (cutaneous*[Title/Abstract])	812,419

	#3	(lesion*[Title/Abstract]) OR (swab[Title/Abstract]) OR (sample[Title/Abstract])	1,949,255
	#4	#1 AND #2 AND #3	173
**Scopus**			
	#1	(TITLE-ABS(mpox)) OR (TITLE-ABS(monkeypox)) OR (TITLE-ABS(mpxv))	2,400
	#2	(TITLE-ABS(skin)) OR (TITLE-ABS(cutaneous*))	1,006,876
	#3	(TITLE-ABS(lesion*)) OR (TITLE-ABS(swab)) OR (TITLE-ABS(sample))	6,140,713
	#4	#1 AND #2 AND #3	145
**Web of Science**			
	#1	((TI=mpox OR AB=mpox)) OR ((TI=monkeypox OR AB=monkeypox)) OR ((TI=mpxv OR AB=mpxv))	1,729
	#2	((TI=skin OR AB=skin)) OR ((TI=cutaneous* OR AB=cutaneous*))	664,566
	#3	((TI=lesion* OR AB=lesion*)) OR ((TI=swab OR AB=swab)) OR ((TI=sample OR AB=sample))	4,262,363
	#4	#1 AND #2 AND #3	100

Annexure S4: Risk of Bias assessment of included studies using National Institute of Health (NIH) tools. *a) for Case series; b) for Cross-sectional studies*

Annexure 4a

Author (YOP)	Q1	Q2	Q3	Q4	Q5	Q6	Q7	Q8	Q9	Overall Quality
**Loconsole D et al., (2022)**[33]	Y	Y	Y	NA	NA	Y	NA	Y	Y	Good
**Palich R et al., (2023)**[15]	Y	Y	Y	NA	NA	Y	NA	Y	Y	Good
**Peiró-Mestres A et al., (2022)**[16]	Y	Y	Y	NA	NA	Y	NA	Y	Y	Good

YOP: Year of Publication; Y: Yes; N: No; NA: Not Applicable; CD: Cannot Determine; NI: No Information; NIH: National Institute of Health

Q1: Was the study question or objective clearly stated?
Q2: Was the study population clearly and fully described, including a case definition?
Q3: Were the cases consecutive?
Q4: Were the subjects comparable?
Q5: Was the intervention clearly described?
Q6: Were the outcome measures clearly defined, valid, reliable, and implemented consistently across all study participants?
Q7: Was the length of follow-up adequate?
Q8: Were the statistical methods well-described?
Q9: Were the results well-described?

Annexure 4b

Author (YOP)	Q1	Q2	Q3	Q4	Q5	Q6	Q7	Q8	Q9	Q10	Q11	Q12	Q13	Q14	Quality rating
**Hasso M et al. (2022)**[35]	Y	Y	Y	Y	N	NA	NA	NA	NA	NA	Y	NA	NA	NA	Good
**Mailhe et al. (2022)**[32]	Y	Y	Y	Y	N	NA	NA	NA	NA	NA	Y	NA	NA	NA	Good
**Ouafi M et al. (2022)**[29]	Y	Y	Y	Y	N	NA	NA	NA	NA	NA	Y	NA	NA	NA	Good
**Tarín-Vicente EJ et al. (2022)**[34]	Y	Y	Y	Y	N	NA	NA	NA	NA	NA	Y	NA	NA	NA	Good
**Ubals M et al. (2022)**[30]	Y	Y	Y	Y	N	NA	NA	NA	NA	NA	Y	NA	NA	NA	Good
**Veintimilla C et al. (2022)**[31]	Y	Y	Y	Y	N	NA	NA	NA	NA	NA	Y	NA	NA	NA	Good

Year of Publication; Y: Yes; N: NO; NA: Not Applicable; CD: Cannot Determine; NI: No Information; NIH: National Institute of Health

Q1: Was the research question or objective in this paper clearly stated?
Q2: Was the study population clearly specified and defined?
Q3: Was the participation rate of eligible persons at least 50%?
Q4: Were all the subjects selected or recruited from the same or similar populations (including the same time period)? Were inclusion and exclusion criteria for being in the study prespecified and applied uniformly to all participants?
Q5: Was a sample size justification, power description, or variance and effect estimates provided?
Q6: For the analyses in this paper, were the exposure(s) of interest measured prior to the outcome(s) being measured?
Q7: Was the timeframe sufficient so that one could reasonably expect to see an association between exposure and outcome if it existed?
Q8: For exposures that can vary in amount or level, did the study examine different levels of the exposure as related to the outcome (e.g., categories of exposure, or exposure measured as continuous variable)?
Q9: Were the exposure measures (independent variables) clearly defined, valid, reliable, and implemented consistently across all study participants?
Q10: Was the exposure(s) assessed more than once over time?
Q11: Were the outcome measures (dependent variables) clearly defined, valid, reliable, and implemented consistently across all study participants?

Q12: Were the outcome assessors blinded to the exposure status of participants?
Q13: Was loss to follow-up after baseline 20% or less?
Q14: Were key potential confounding variables measured and adjusted statistically for their impact on the relationship between exposure(s) and outcome(s)?

Annexure S5: Bubble plot demonstrating meta-regression based upon the average age of the participants

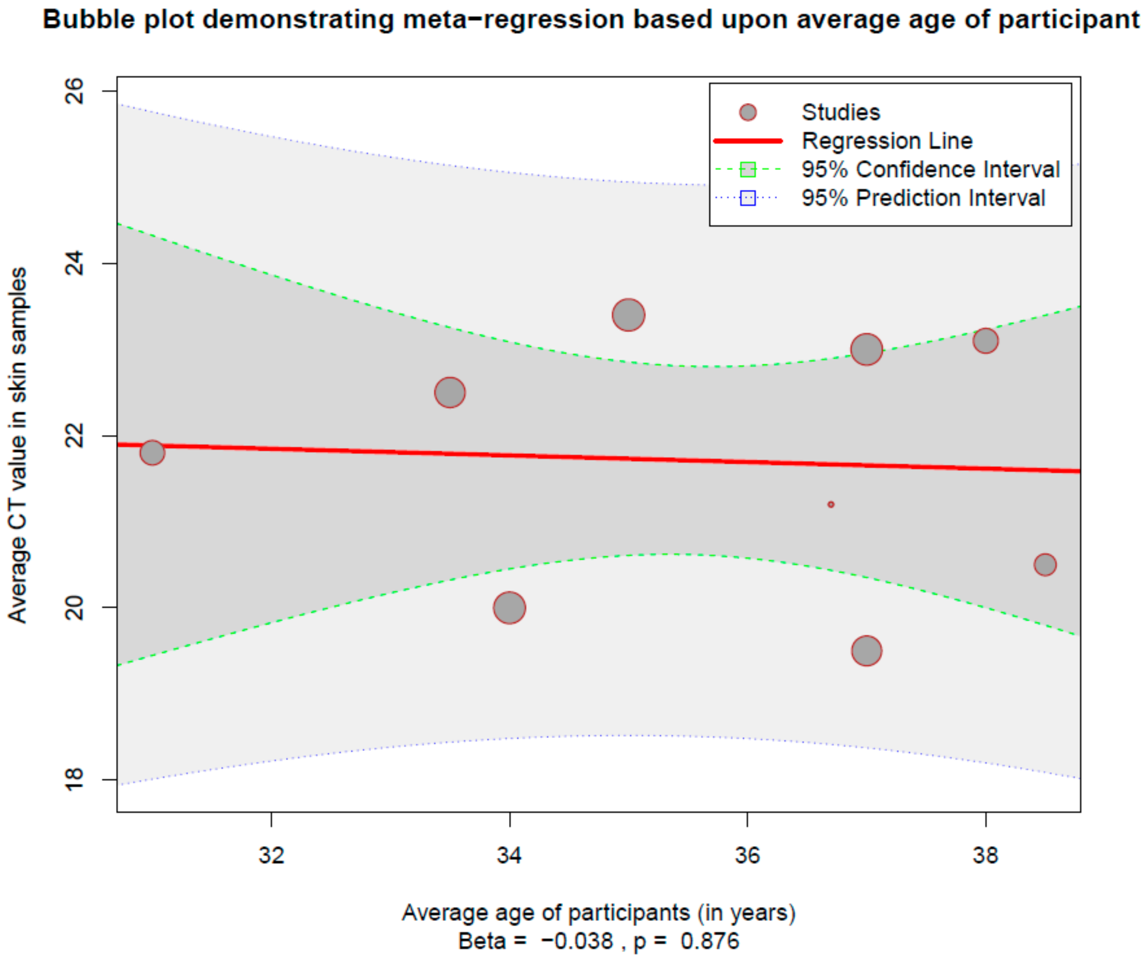

Annexure S6: Bubble plot demonstrating meta-regression based upon the sex distribution of the participants

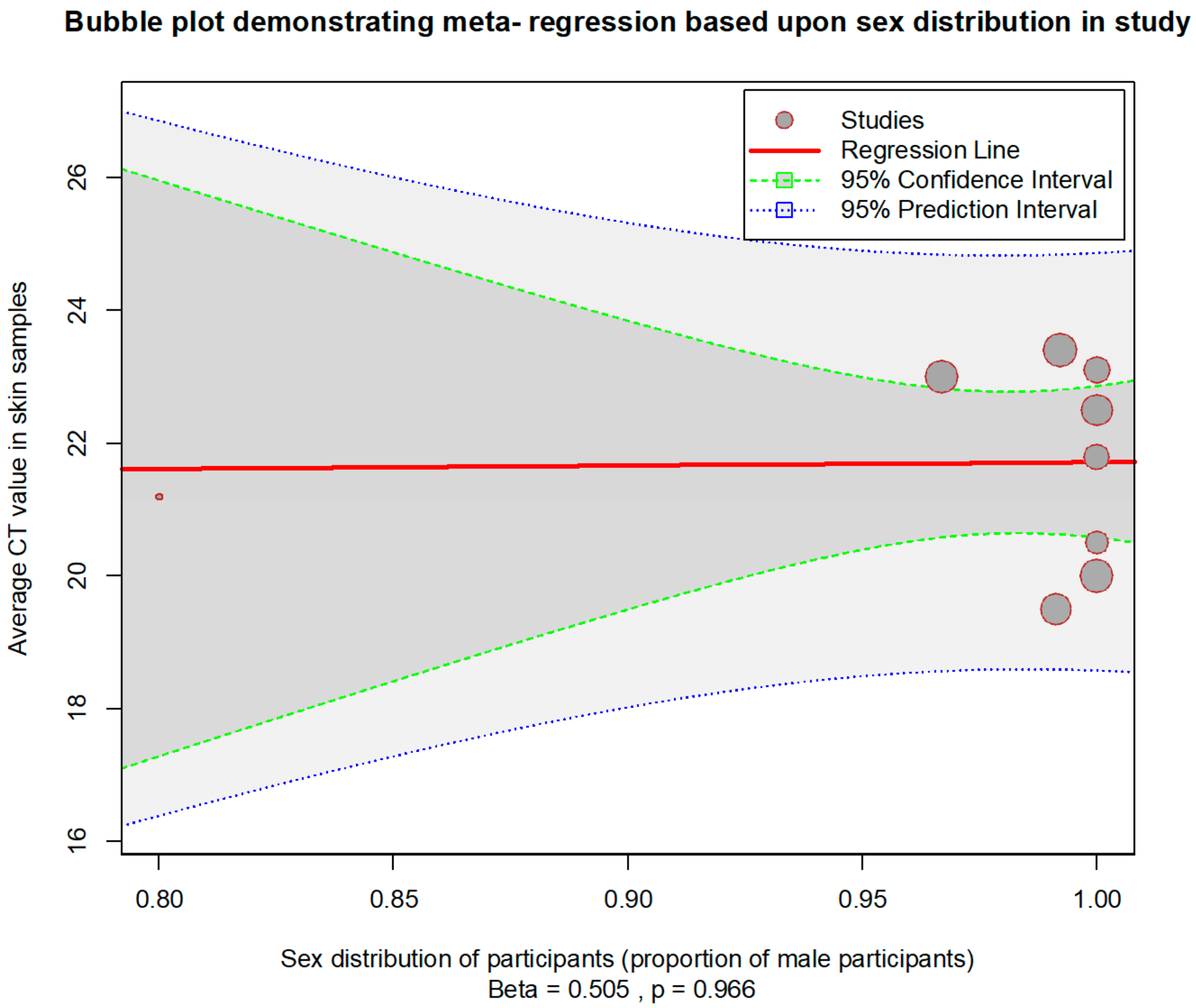

Annexure S7: a-d) Clustering to identify influence and heterogeneity contributed by studies; e) Influence plot to visualize influence; f) Visualization of heterogeneity contributed by the studies plotted against pooled estimate

a.

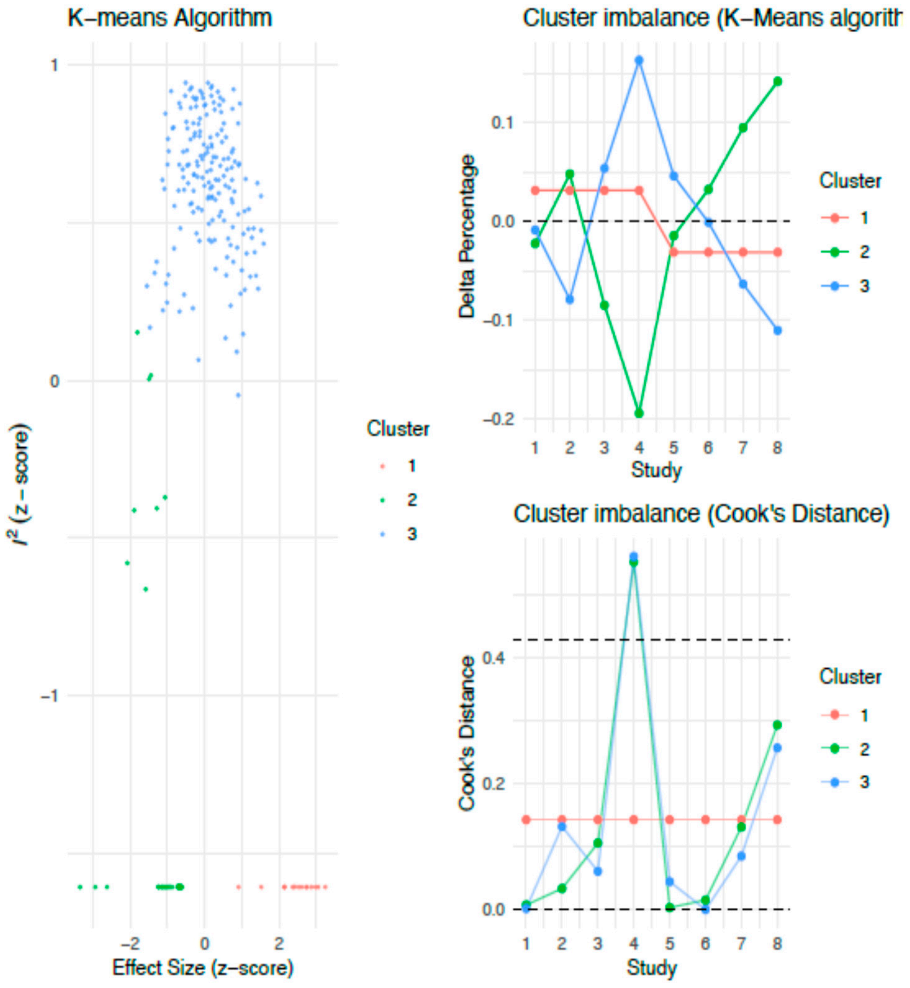

b.

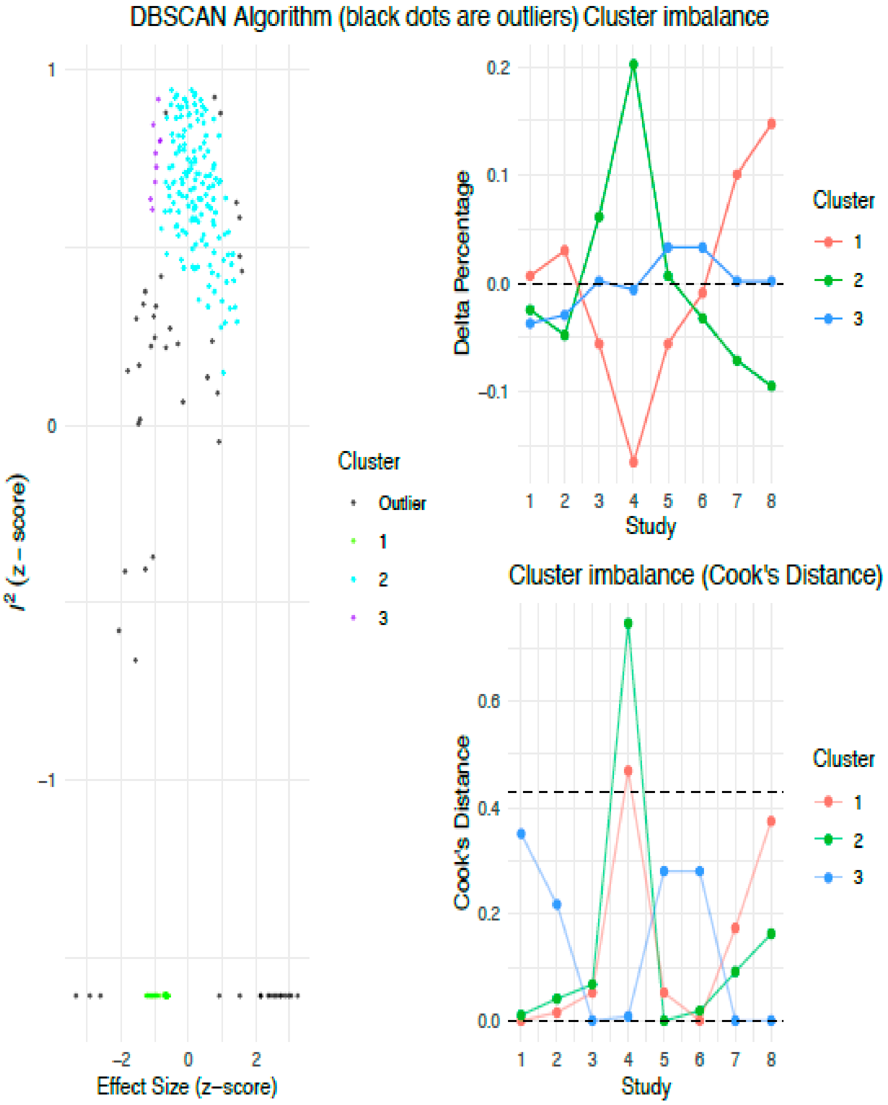

DBSCAN Algorithm (black dots are outliers)
Cluster imbalance
Cluster imbalance (Cook's Distance)
I^2 (z − score)
Effect Size (z−score)
Cluster
Outlier
1
2
3
Delta Percentage
Cook's Distance
Study

c.

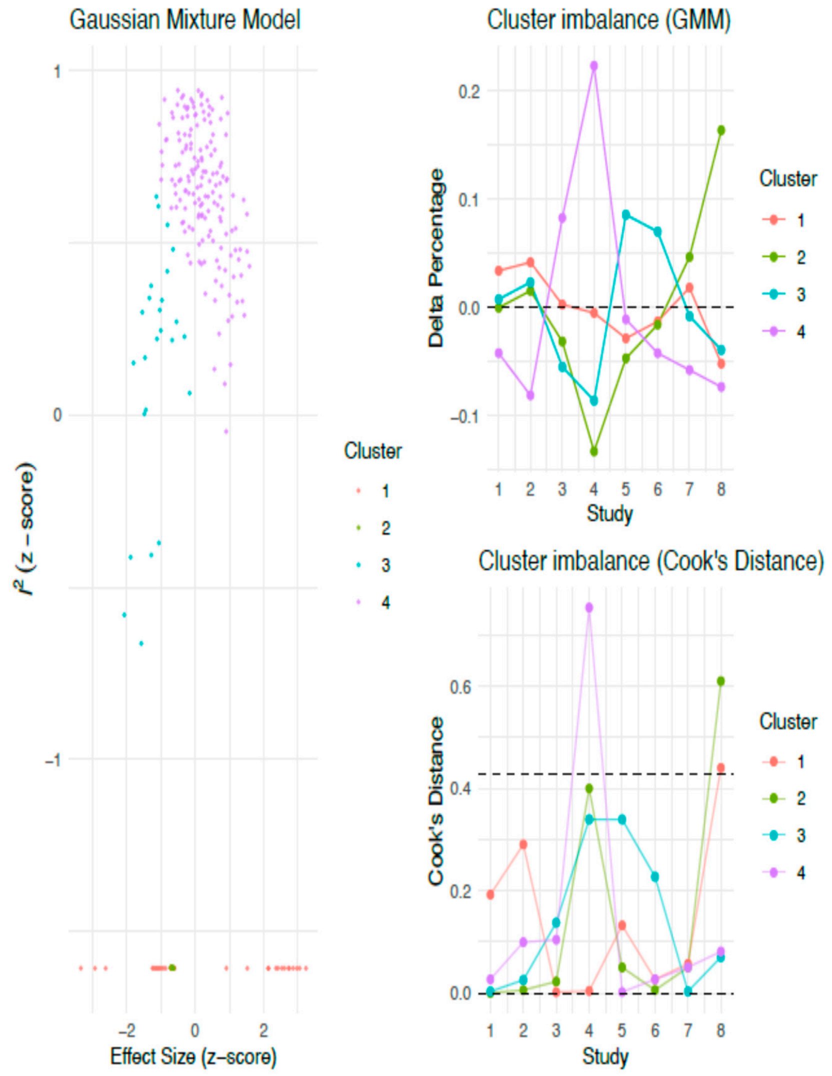

Gaussian Mixture Model
I^2 (z − score)
Effect Size (z−score)
Cluster
1
2
3
4
Cluster imbalance (GMM)
Delta Percentage
Study
Cluster imbalance (Cook's Distance)
Cook's Distance
Study

d.

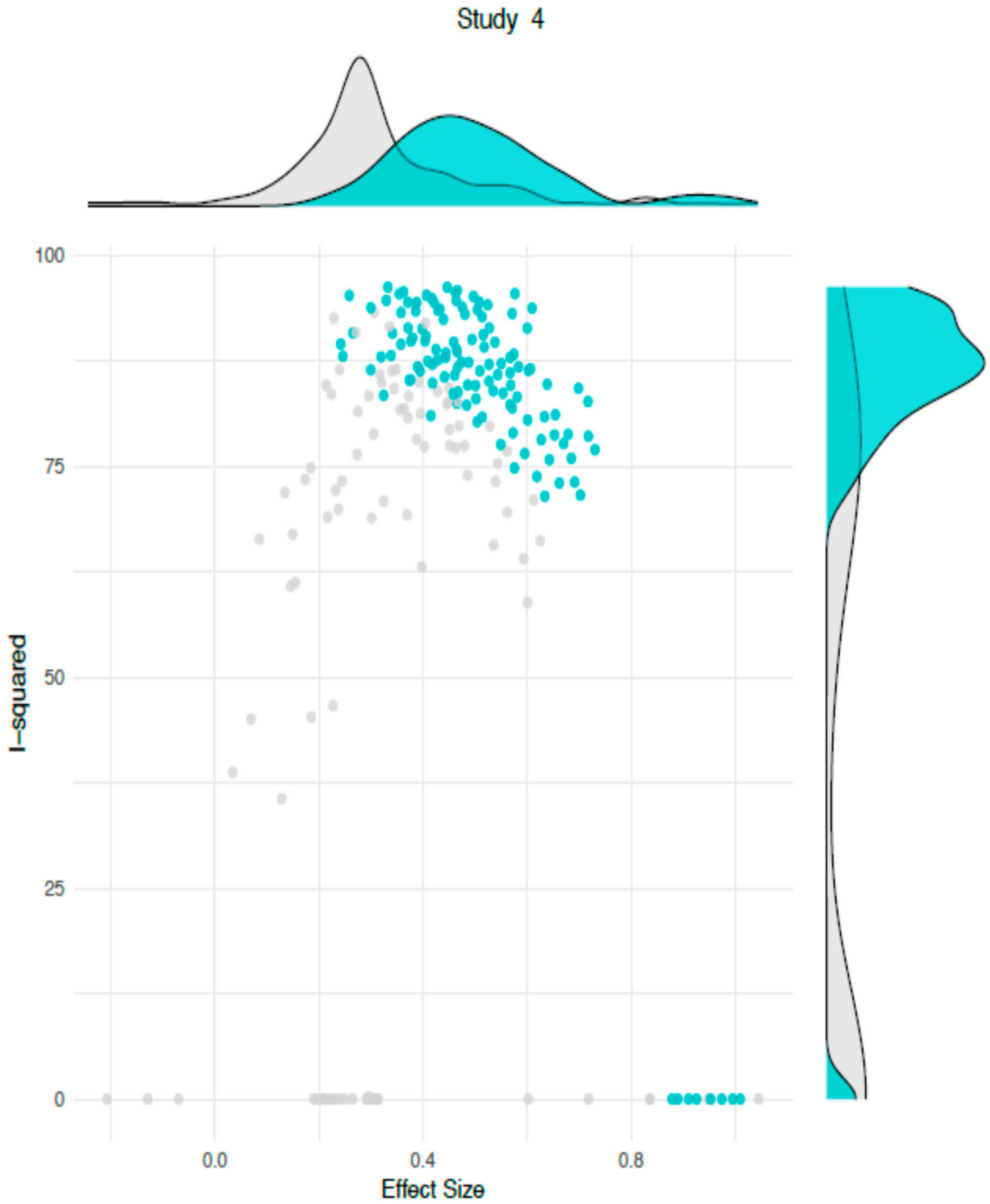

e.

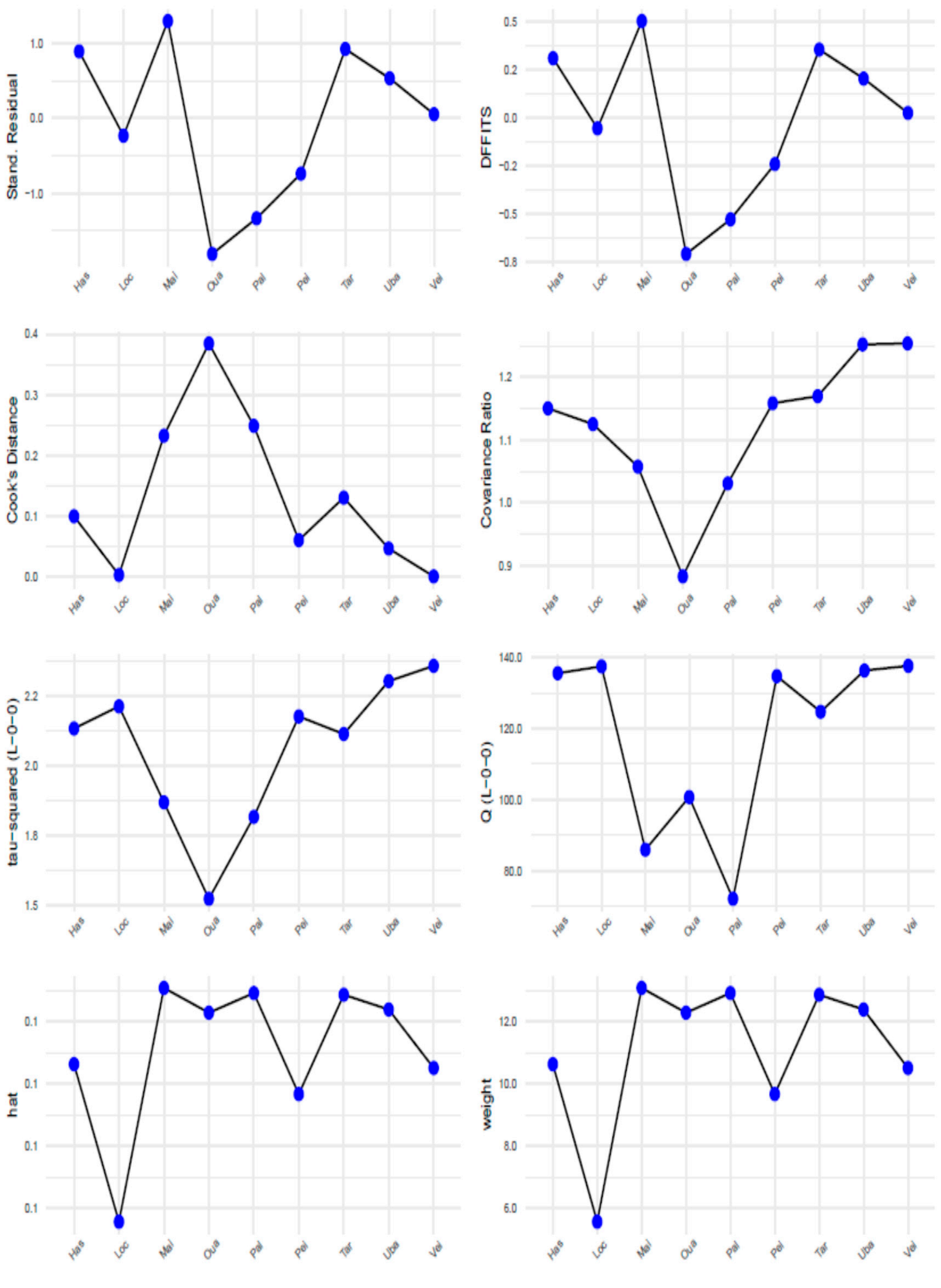
Stand. Residual
DFFITS
Cook's Distance
Covariance Ratio
tau−squared (L−0−0)
Q (L−0−0)
hat
weight
Has
Loc
Mal
Oua
Pal
Pel
Tar
Uba
Vel

f.

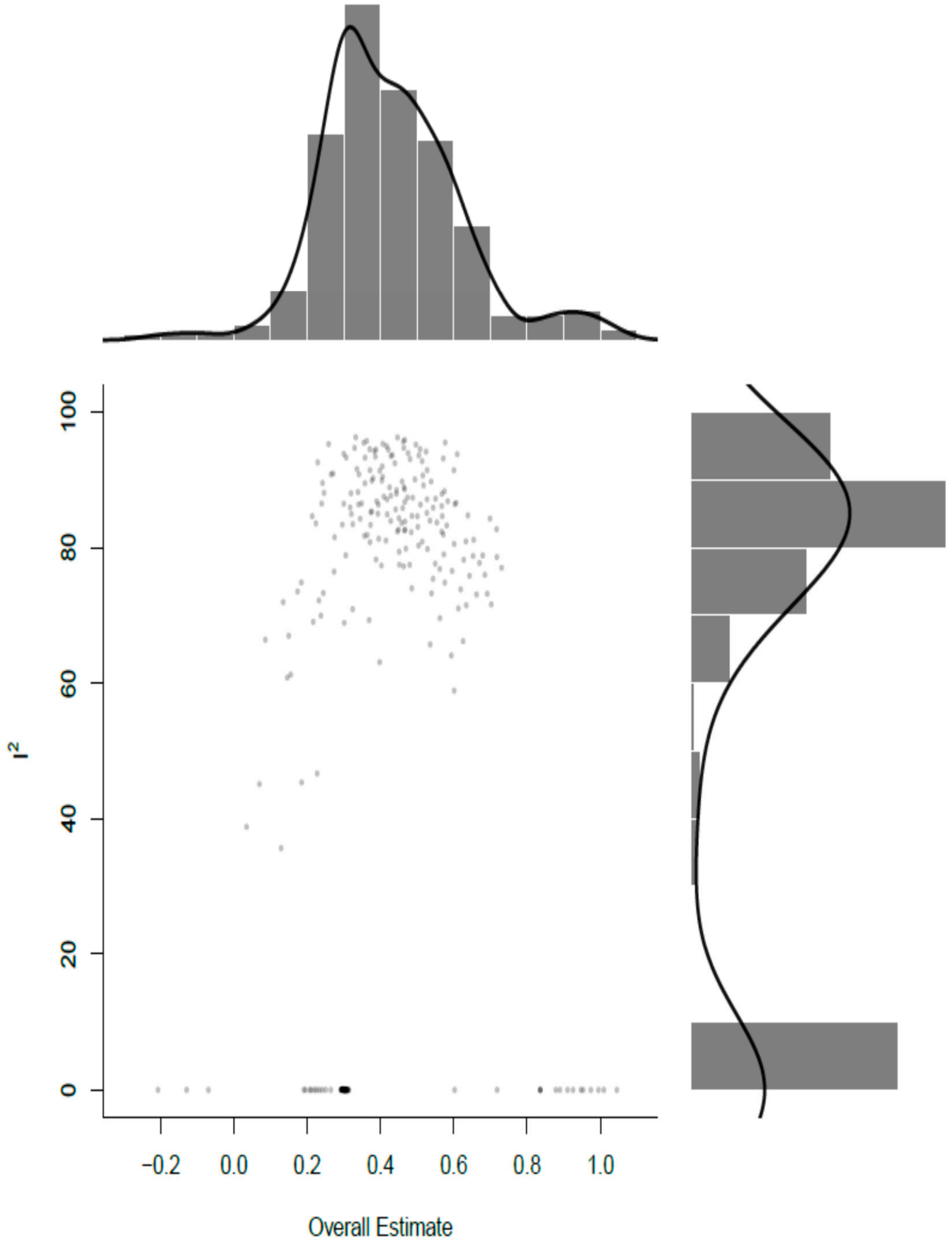

Annexure S8: Leave-one-out meta-analyses for each study showing the effect on the pooled estimate

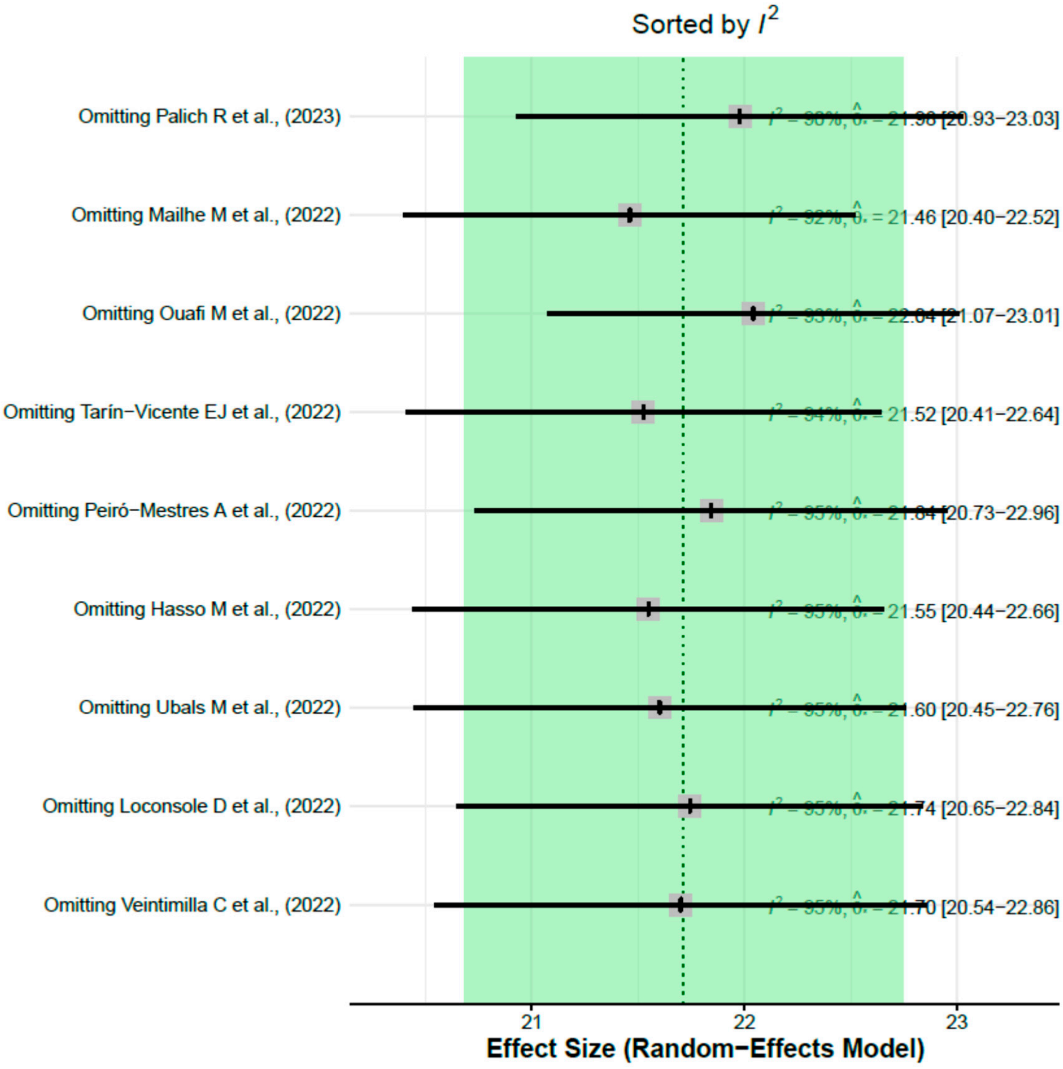

Annexure S9: Funnel plot to demonstrate publication bias and small-study effects

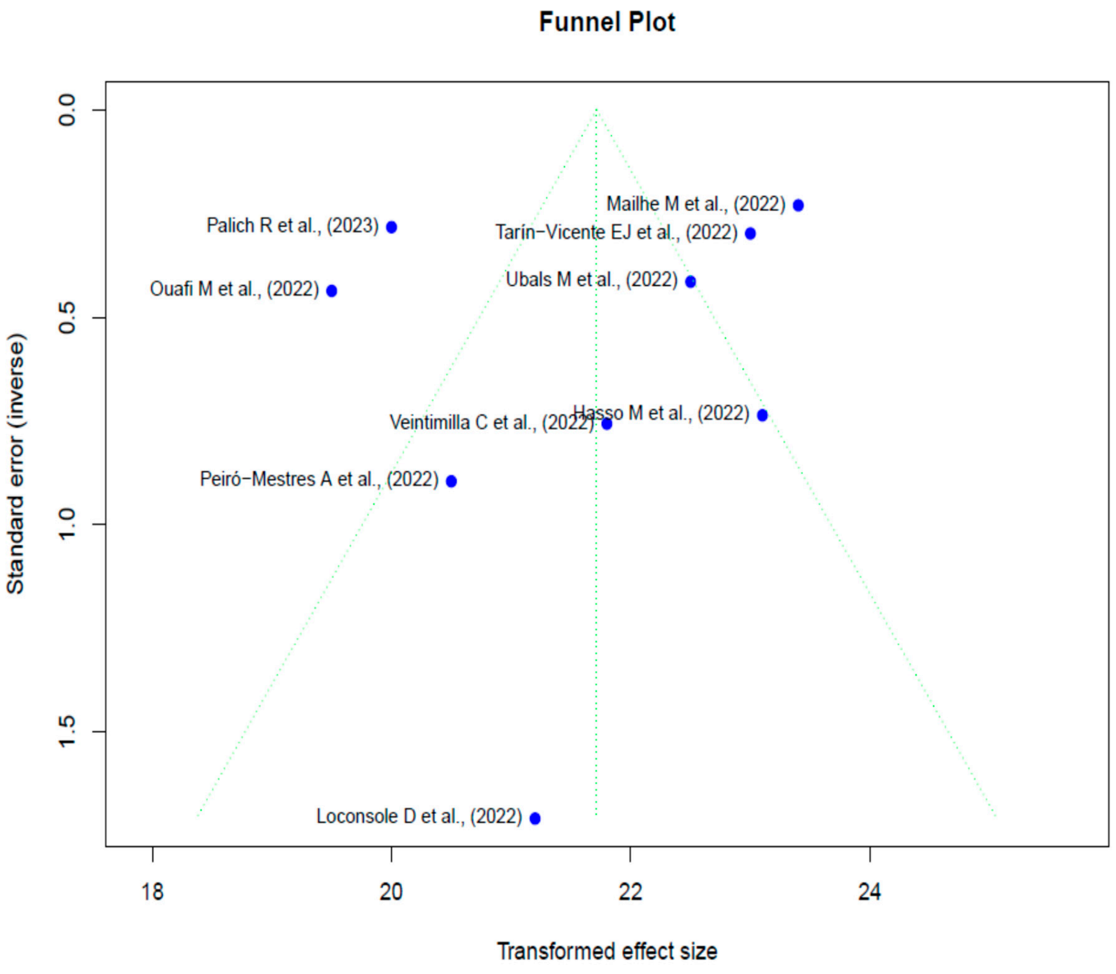

Supplement: Supplementary file 1 [file viruses-15-01386-s001.zip › viruses-2374531-supplementary.pdf]
